# Longitudinal associations between post-traumatic stress and post-traumatic growth among older adults 11 years after a disaster

**DOI:** 10.1017/S2045796024000362

**Published:** 2024-06-26

**Authors:** Hiroyuki Hikichi, Kanako Taku, Jun Aida, Katsunori Kondo, Ichiro Kawchi

**Affiliations:** 1 Kitasato University School of Medicine, Sagamihara, Kanagawa, Japan; 2 Department of Psychology, Oakland University, Rochester, MI, USA; 3 Department of Oral Health Promotion, Graduate School of Medical and Dental Sciences, Tokyo Medical and Dental University, Bunkyo, Tokyo, Japan; 4 Center for Preventive Medical Sciences, Chiba University, Chuo, Chiba, Japan; 5 Center for Gerontology and Social Science, National Center for Geriatrics and Gerontology, Obu, Aichi, Japan; 6 Department of Social and Behavioral Sciences, Harvard T.H. Chan School of Public Health, Boston, MA, USA

**Keywords:** marginal structuralmodels, natural disasters, post-traumatic growth, post-traumatic stress, older individuals

## Abstract

**Aims:**

Previous studies have reported inconsistent findings regarding the association between post-traumatic stress (PTS) and post-traumatic growth (PTG). Three major issues could account for this inconsistency: (1) the lack of information about mental health problems before the disaster, (2) the concept of PTG is still under scrutiny for potentially being an illusionary perception of personal growth and (3) the overlooking of PTS comorbidities as time-dependent confounding factors. To address these issues, we explored the associations of PTS and PTG with trauma-related diseases and examined the association between PTS and PTG using marginal structural models to address time-dependent confounding, considering pre-disaster covariates, among older survivors of the 2011 Japan Earthquake and Tsunami.

**Methods:**

Seven months before the disaster, the baseline survey was implemented to ask older adults about their health in a city located 80 km west of the epicentre. After the disaster, we implemented follow-up surveys approximately every 3 years to collect information about PTS and comorbidities (depressive symptoms, smoking and drinking). We asked respondents about their PTG in the 2022 survey (*n* = 1,489 in the five-wave panel data).

**Results:**

PTG was protectively associated with functional disability (coefficient −0.47, 95% confidence interval (CI) −0.82, −0.12, *P* < 0.01) and cognitive decline assessed by trained investigators (coefficient −0.07, 95% CI −0.11, −0.03, *P* < 0.01) and physicians (coefficient −0.06, 95% CI −0.11, −0.02, *P* < 0.01), while PTS was not significantly associated with them. Severely affected PTS (binary variable) was associated with higher PTG scores, even after adjusting for depressive symptoms, smoking and drinking as time-dependent confounders (coefficient 0.35, 95% CI 0.24, 0.46, *P* < 0.01). We also found that an ordinal variable of the PTS score had an inverse U-shaped association with PTG.

**Conclusion:**

PTG and PTS were differentially associated with functional and cognitive disabilities. Thus, PTG might not simply be a cognitive bias among survivors with severe PTS. The results also indicated that the number of symptoms in PTS had an inverse U-shaped association with PTG. Our findings provided robust support for the theory of PTG, suggesting that moderate levels of psychological struggles (i.e., PTS) are essential for achieving PTG, whereas intense PTS may hinder the attainment of PTG. From a clinical perspective, interventions that encourage social support could be beneficial in achieving PTG by facilitating deliberate rumination.

## Introduction

Older people are vulnerable to the impacts of natural disasters. Disaster-related traumatic experiences persistently affect the physical, mental and cognitive health as well as the well-being of older survivors (Shiba *et al.*, 2022).

Nevertheless, despite the vulnerability of older victims of traumatic events, a subset of them may attain personal growth through struggling with adversities (Greenblatt-Kimron, 2021; Kadri *et al.*, 2022). Post-traumatic growth (PTG) is a positive psychological change that comprises five major dimensions: improved relationships with others, increased personal strength, identification of new possibilities, positive spiritual changes and increased appreciation of life (Tedeschi and Calhoun, 2004). A meta-analysis demonstrated that adult survivors of earthquakes tend to exhibit moderate levels of PTG (Amiri *et al.*, 2021).

Experiencing traumatic events exhibits complicated relationships with psychological resilience, as it is associated not only with PTG but also with post-traumatic stress (PTS). Most research has demonstrated a positive association (linear or inverted U-shaped) between them (Shakespeare-Finch and Lurie-Beck, 2014), while some findings have also suggested an inverted linear or non-significant relationship (Kadri *et al.*, 2022).

Previous studies have highlighted three issues. First, the absence of pre-disaster information is a limitation in most studies, given that both PTS and PTG can be influenced by the presence of psychopathology preceding exposure to disaster (Long *et al.*, 2021). Second, the concept of PTG continues to be debated. Some research has suggested that PTG might be a form of cognitive bias among survivors – for instance, social desirability bias, positive attrition bias or the illusory perception of personal growth as a compensatory response to trauma (Gower *et al.*, 2022). Third, ignoring time-dependent confounding can result in biased estimates of associations (Robins *et al.*, 2000). For example, as shown in Figure S1, PTS at time 1 is significantly associated with increased risks of comorbidities including depression and substance use at time 2 (Flory and Yehuda, 2015; Sareen, 2014) which in turn could be linked to severe PTS at the same survey wave (Long *et al.*, 2021; Koenen *et al.*, 2005; Stewart, 1996), and a certain level of PTG at time 4 in a subsequent wave (Stump and Smith, 2008). This scenario of time-dependent confounding cannot be adequately addressed by conventional methods such as sample restriction, stratification or covariance adjustment (Mansournia *et al.*, 2017).

To address these issues, we documented the 11.5-year post-disaster follow-up of a cohort of community-dwelling older adults who survived the 2011 Great East Japan Earthquake and Tsunami. By coincidence, we had collected pre-disaster information on the health and lifestyle of the respondents 7 months prior to the disaster. This natural experimental design provides us with the opportunity to pursue two objectives: (1) exploring the associations between PTG and PTS with trauma-related diseases, taking into account pre-disaster information and (2) examining the relationship between PTS and PTG using marginal structural models to address time-dependent confounding.

## Methods

### Study participants

The Japan Gerontological Evaluation Study (JAGES) was established in 2010, sampling residents aged 65 or older nationwide. One of the field sites of the JAGES cohort is based in the Iwanuma City (total population of 44,187 in 2010). We mailed questionnaires to all older residents in August 2010 (*n* = 8,576), using the city’s residential register. The survey covered personal characteristics, lifestyle and health, with a 59.0% response rate (*n* = 5,058), which is comparable to other surveys of community-dwelling residents (Santos-Eggimann *et al.*, 2009).

The 11 March 2011 earthquake and tsunami severely affected Iwanuma City, located 80 km west of the epicenter. The disaster caused 180 deaths, damaged 5,542 homes and flooded 48% of Iwanuma’s land (Figure S2).

We conducted follow-up surveys of survivors four times in 2013, 2016, 2019 and 2022, gathering information on health status, lifestyle and housing types after the disaster. We inquired about PTG only in the 2022 survey.

**Figure 1.**
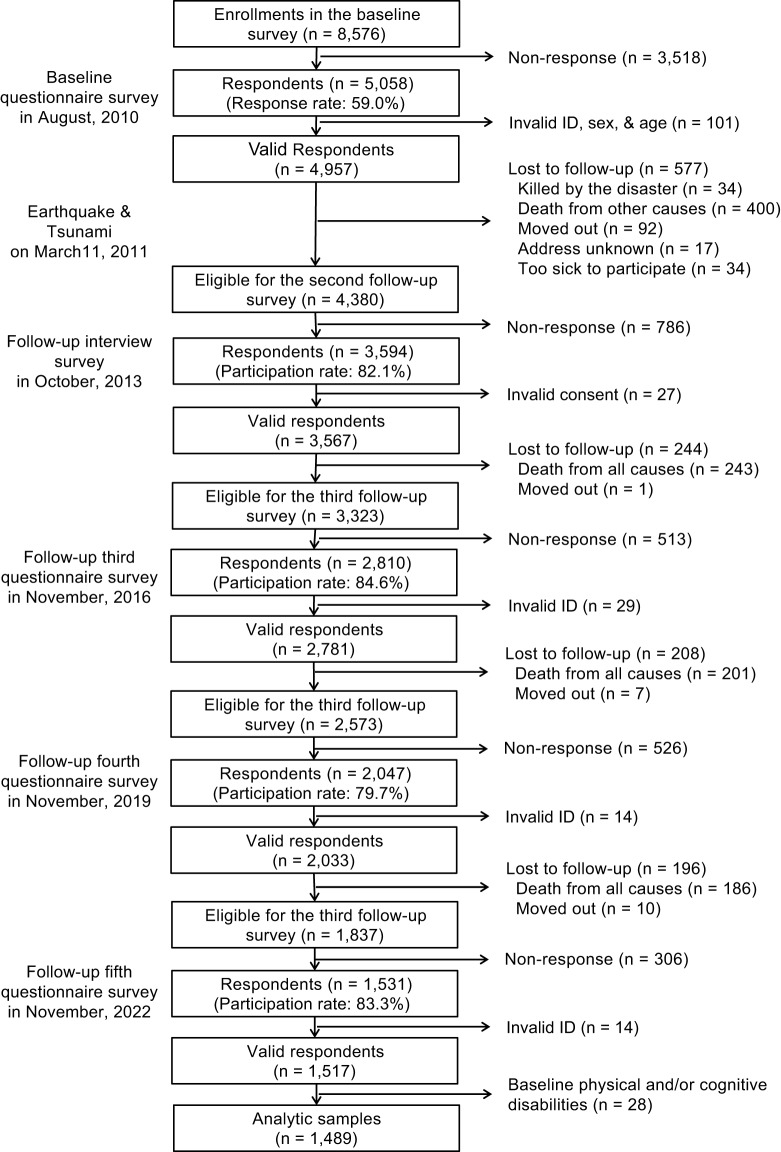
Participant flow in this study.

The detailed flow chart of the analytic sample is presented in Fig. 1. The respondents with physical and cognitive disabilities at the baseline (*n* = 28) were excluded from this study because they are particulary vulnerable in the aftermath of disasters (Aldrich and Benson, 2008), which may affect both PTS and PTG as confounders. Finally, the analytic sample was 1,489 respondents.

The survey protocol was reviewed and approved by the Ethics Committees on Research of Human Subjects at the Harvard T.H. Chan School of Public Health, Tohoku University, Nihon Fukushi University and Chiba University. Informed consent was obtained at the time of survey collection.

### Explanatory variables

PTS was assessed using the Screening Questionnaire for Disaster-Related Mental Health (SQDRMH) (Fujii *et al.*, 2007), originally developed and psychometrically validated in the aftermath of the 1995 Hanshin–Awaji earthquake in Japan. The instrument was specifically developed for screening disaster-related mental disorders of older survivors and has been psychometrically validated against the Japanese-language version of the Clinician-Administered PTSD Scale (CAPS) (Blake *et al.*, 1995).

In the surveys, we asked respondents: ‘People who have experienced the 2011 Japan Earthquake and Tsunami often report that their lives have changed dramatically, and they are constantly under various kinds of stress. Have you experienced any of the symptoms listed below in the past month?’, and then asked their symptoms using the following nine questions: ‘Do you have trouble falling asleep or sleeping through the night?’, ‘Do you have nightmares about the event?’, ‘Do you feel irritable?’, ‘Do you feel that you are hypersensitive to small noises or tremors?’, ‘Do you avoid places, people and topics related to the event?’, ‘Do you think about the event when you do not want to?’, ‘Do you have trouble enjoying things you used to enjoy?’, ‘Do you get upset when something reminds you of the event?’ and ‘Do you notice that you are making an effort to try not to think about the event or are trying to forget it?’.

The nine items exhibited a reasonable internal consistency (Cronbach’s α = 0.74). Following previous studies suggesting a non-linear association between PTS and PTG, we categorized PTS as binary: 1 for severely affected (6 to 9 points) and 0 for moderately or slightly affected (0 to 5 points) (Fujii *et al.*, 2007). Additionally, we used ordinal variables and, in a sensitivity analysis, a continuous variable for PTS.

### Outcome variable

Our primary outcomes were obtained from objective assessments of functional, cognitive and physical disabilities, in addition to PTG.

1. Functional disability. The level of functional disability was obtained from the Japanese Long-Term Care Insurance (LTCI) database. Since 2001, the Japanese government has implemented a national insurance scheme for older individuals requiring long-term care (e.g., home helpers). Applicants are classified into one of seven care levels through a standardized multistep assessment of functional and cognitive disabilities conducted by trained investigators and physicians. These levels correspond to the severity of their disabilities and the estimated hours of home care needed each week (e.g., bathing, dressing, cleaning the house and preparing meals) (Tamiya *et al.*, 2011).
2. Cognitive disability. Trained investigators assess applicants’ cognitive function (e.g., short-term memory, orientation and communication) and mental and behavioural disorders (e.g., delusions of persecution and confabulation) using a standardized protocol implemented during in-home assessment. Following this assessment, applicants are classified into one of seven levels, ranging from 1 (experiencing some cognitive deficits but otherwise almost completely independent) to 7 (requiring constant treatment in a specialized medical facility), based on the severity of their cognitive disability. Additionally, a panel of physicians independently assess the level of cognitive disability of applicants to determine their care requirements (Olivares-Tirado and Tamiya, 2014).
3. Physical disability. Under the LTCI scheme, trained investigators assess applicants’ activities of daily living and instrumental activities of daily living, and classify applicants into one of eight levels (1: Suffers from some level of disability but is able to function independently for the most part and can manage outings alone using public transportation, to level 8: Spends the whole day in bed and requires assistance in toileting, preparing meals, changing clothes and even turning over in bed).
4. PTG. PTG was measured using the Short Form of the Expanded Version of the Posttraumatic Growth Inventory (PTGI-X-SF-J) (Oshiro *et al.*, 2023). This scale assessed five dimensions of PTG: improved relationship with others, increased personal strength, identification of new possibilities, positive spiritual changes and increased appreciation of life.

We instructed participants ‘Please tell us to what extent your daily life has changed as a result of experiencing the 2011 Japan Earthquake and Tsunami’ and asked them the following questions. Improved relationship with others was measured using the following two items: ‘I have a greater sense of closeness with others’ and ‘I more clearly see that I can count on people in times of trouble.’ Increased personal strength was measured using the following two questions: ‘I know better how to handle difficulties’ and ‘I discovered that I’m stronger than I thought I was.’ The following two items were used to assess the identification of new possibilities: ‘New opportunities are available which wouldn’t have been otherwise’ and ‘I established a new path for my life.’ Positive spiritual changes were measured using the following two questions: ‘I feel more connected with all of existence’ and ‘I have a greater sense of harmony with the world.’ We used the following two questions to measure the increased appreciation of life: ‘I have a greater appreciation for the value of my own life’ and ‘I can better appreciate each day.’ Responses were ordered along a six-point Likert scale (1: not at all, 6: very strong).

As shown in Figure S3, we implemented a confirmatory factor analysis to check the construct validity of PTG measured by 10 items and found a reasonable five-factor solution with acceptable goodness-of-fit indices (comparative fit index 0.981, root-mean-square error of approximation 0.079 and standardized-root-mean-square residual 0.025). We calculated the arithmetical mean of the responses to the 10 items to create a total PTG score, which indicated good internal consistency (Cronbach’s α = 0.94).

### Covariates

Based on previous studies (Hikichi *et al.*, 2021, 2016), we chose several baseline demographic variables as potential confounding variables for the association of PTS and PTG: age, sex, equivalized household income, educational attainment, divorce or bereavement and employment status.

For equivalized household income, we divided the household’s gross income by the square root of the number of household members (OECD, 2013).

Based on previous findings about risk factors for PTG, we also adjusted for the number of adverse life events (Fraus *et al.*, 2023) at the baseline. Participants were asked whether they had experienced any of the following events within the past year: resignation, living alone, financial difficulty, loss of a spouse, loss of relatives or friends and serious illnesses. We then totalled the number of adverse life events reported.

We also chose depressive symptoms, and smoking and drinking habits in each survey as comorbidities of PTS. Depressive symptoms were measured by the Geriatric Depression Scale-15 (GDS-15; Sugishita *et al.*, 2017). The baseline GDS-15 items showed good internal consistency, with a Cronbach’s α of 0.79. We created a binary variable, dichotomized at a clinically validated cutoff point, to demonstrate that a higher level of depressive symptoms is correlated with PTG. This variable distinguished between lower risk (4 points and under) and higher risk (5 points and over) (Weintraub *et al.*, 2006). In a sensitivity analysis, we utilized a continuous variable of the GDS-15 score.

We also considered post-disaster housing. Immediately after the disaster, survivors who lost their homes had options, including moving into disaster-relief housing, renting apartments or purchasing new housing. When the disaster-relief housing closed in April 2016 and Iwanuma City opened permanent housing, survivors had another opportunity to choose from government housing, market rentals or home purchases. The variable indicates four housing categories: 1 = no relocation, 2 = government-provided housing, 3 = apartments in the open rental market and 4 = newly purchased housing.

### Statistical analysis

First, we employed linear regression models to examine the cross-sectional association of PTG and PTS with functional, cognitive and physical disabilities considering baseline (pre-disaster) covariates.

Second, we examined the association between PTS and PTG using three models: (1) adjusting for only baseline covariates, (2) including time-series variables and (3) considering time-dependent confounding and inverse probability weighting (i.e., marginal structural models). In this analysis, we utilized two types of datasets: a five-wave panel (Fig. 1) and imbalanced data consisting of baseline respondents who participated in at least one of the follow-up surveys. Specifically, the imbalanced data included a significant number of attrition cases, which were adjusted for in the analysis.

We estimated the stabilized inverse probability of receiving the treatment (i.e., a higher risk of PTS) *SW*(*t*) and the probability of participating in surveys up to time t *SW*(*c*) to create a pseudo-population to balance the distribution of potential confounders across exposure levels and remained cases (Godin *et al.*, 2012). *SW*(*t*) and *SW*(*c*) are defined as

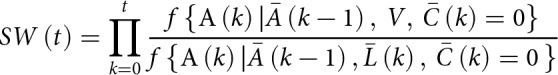


and

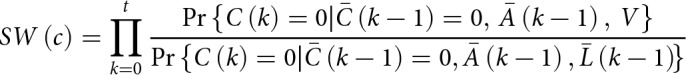


where 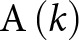 denotes the exposure at year 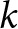, 
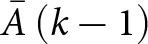
 represents the exposure history prior year 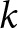, 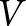 is baseline covariates, 
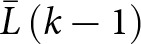
 represents the covariates history including 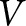, 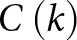 is the incident attrition at year 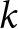 and 
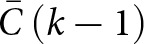
 is participating history until year 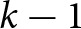.

We estimated only 
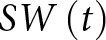
 for the five-panel data because of no attrition cases, while we calculated both 
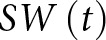
 and 
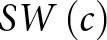
 for the imbalanced data to adjust selection bias due to dropped cases.

Throughout these analyses, we utilized binary and ordinal variables for PTS as previous studies have suggested non-linear associations between PTS and PTG (Kadri *et al.*, 2022). Specifically, the ordinal variable of PTS was likely a suitable choice to understand their unique association, especially since respondents with severe PTS exhibited higher proportions of comorbidities, such as severe depressive symptoms and current smoking (Table S1). In a sensitivity analysis, we used a continuous variable for PTS.

We implemented multiple imputation for missing values using the Markov chain Monte Carlo method that assumes missingness at random. Twenty datasets were created, and each result of analyses was combined using the Stata command ‘mi estimate’. All analyses were performed using STATA version 17.0 (STATA Corp LP, College Station, TX, USA).

## Results

Table 1 presents descriptive statistics for the outcomes, explanatory variables and potential covariates. Of the 1,489 respondents, 8.5% were identified as having severely affected PTS in 2013, which rose to 9.3% by 2022, although it was not statistically significant (Cochran–Armitage Trend Test (CA): χ^2^ = 2.121, *P* = 0.15). Moreover, 23.0% of the respondents showed severe depression in 2010, which increased to 27.0% by 2022 (CA: χ^2^ = 17.924, *P* < 0.01). The average PTG score was 3.18 in 2022. On the other hand, the baseline proportions of smokers and drinkers (10.1% and 40.6%, respectively) greatly reduced by 2022 (4.2% and 30.4%, respectively) (CA: χ^2^ = 57.215, *P* < 0.01, for smoking; χ^2^ = 30.423, *P* < 0.01, for drinking).

**Table 1.** Characteristics of the analytic samples in the five-wave panel (*n* = 1,489)

	*n* (%)/mean (SD)				
	2010	2013	2016	2019	2022
Explanatory variables					
Posttraumatic stress, *n* (%)					
Severely affected^a^		126 (8.5)	104 (7.0)	129 (8.7)	139 (9.3)
Missing, *n* (%)		58 (3.9)	162 (10.9)	81 (5.4)	112 (7.5)
Outcome					
Posttraumatic growth, mean (SD)					3.18 (1.11)
Missing, *n* (%)					288 (19.3)
Time-dependent covariates					
Depressive symptoms, *n* (%)					
Severe^b^	342 (23.0)	341 (22.9)	273 (18.3)	357 (24.0)	402 (27.0)
Missing, *n* (%)	146 (9.8)	160 (10.8)	324 (21.8)	164 (11.0)	285 (19.1)
Current smoking, *n* (%)					
Yes	150 (10.1)	121 (8.1)	86 (5.8)	75 (5.0)	63 (4.2)
Missing, *n* (%)	80 (5.4)	10 (0.7)	77 (5.2)	22 (1.5)	21 (1.4)
Current drinking, *n* (%)					
Yes	605 (40.6)	547 (36.7)	524 (35.2)	496 (33.3)	453 (30.4)
Missing, *n* (%)	22 (1.5)	5 (0.3)	94 (6.3)	36 (2.4)	60 (4.0)
Baseline characteristics					
Age, mean (SD)	71.0 (4.66)				
Sex, *n* (%)	842 (56.6)				
Educational attainment (1: < 6 years to 4: ≥13 years), mean (SD)	2.95 (0.72)				
Missing, *n* (%)	43 (2.9)				
Equivalized household income (10,000 JPY), mean (SD)	241.12 (137.34)				
Missing, *n* (%)	179 (12.0)				
Divorce/bereavement, *n* (%)	294 (19.7)				
Missing, *n* (%)	29 (2.0)				
Working, *n* (%)	313 (21.0)				
Missing, *n* (%)	127 (8.5)				
The number of adverse life events, mean (SD)	0.66 (0.79)				
Missing, *n* (%)	67 (4.5)				
Disaster-related experiences					
Housing status after the disaster^c^					
No relocation, *n* (%)		1,389 (93.3)			1,344 (90.3)
Government-provided housing, *n* (%)		29 (2.0)			14 (0.9)
Apartment in the open-rental market, *n* (%)		12 (0.8)			13 (0.9)
Newly purchased housing, *n* (%)		21 (1.4)			50 (3.4)
Missing, *n* (%)		38 (2.6)			68 (4.6)

Abbreviation: JPY, Japanese yen

a Severely affected PTS was defined as a score of ≥6 points of Screening Questionnaire for Disaster-Related Mental Health.

b Severe depressive symptoms were defined as a score of ≥5 points of Geriatric Depression Scale-15.

c We assumed that the housing status in 2016 and 2019 was the same as the status in 2022 because victims relocated to a permanent housing complex from the disaster relief housing community in April 2016.

As shown in Table S1, respondents who had severe PTS exhibited higher proportions of severe depressive symptoms than those with milder PTS across the four follow-up surveys (48.4% vs. 20.5% in 2013, 35.6% vs. 16.9% in 2016, 50.4% vs. 21.1% in 2019 and 51.1% vs. 24.7% in 2022). They also showed slightly higher rates of smoking in the latter two surveys (5.4% vs. 5.0% in 2019 and 5.0% vs. 4.4% in 2022).

Table 2 presents the result of linear regression models. PTG was protectively associated with functional disability (coefficient −0.47, 95% confidence interval (CI) −0.82, −0.12, *P* < 0.01) and cognitive decline assessed by trained investigators (coefficient −0.07, 95% CI −0.11, −0.03, *P* < 0.01) and physicians (coefficient −0.06, 95% CI −0.11, −0.02, *P* < 0.01), but not significantly associated with physical disability (coefficient −0.02, 95% CI −0.17, 0.13, *P* = 0.76). On the other hand, PTS was not significantly associated with those outcomes (coefficient −0.01, 95% CI −0.19, 0.18, *P* = 0.92; coefficient −0.01, 95% CI −0.03, 0.02, *P* = 0.67; coefficient −0.01, 95% CI −0.03, 0.01, *P* = 0.30; and coefficient −0.02, 95% CI −0.09, 0.05, *P* = 0.54, respectively).

**Table 2.** Associations of PTG and PTS with physical and cognitive impairments in the year 2022

	Outcomes in 2022							
	Functional dependence		Cognitive decline assessed by trained investigators		Cognitive decline assessed by physicians		Physical disabilities	
	Coefficient (95% CI)	*P*	Coefficient (95% CI)	*P*	Coefficient (95% CI)	*P*	Coefficient (95% CI)	*P*
Explanatory variables in 2022								
Model1: PTG	−0.47 (−0.82, −0.12)	<0.01	−0.07 (−0.11, −0.03)	<0.01	−0.06 (−0.11, −0.02)	<0.01	−0.02 (−0.17, 0.13)	0.76
Model 2: PTS	−0.01 (−0.19, 0.18)	0.92	−0.01 (−0.03, 0.02)	0.67	−0.01 (−0.03, 0.01)	0.30	−0.02 (−0.09, 0.05)	0.54

Abbreviations: CI, confidence interval; PTS, post-traumatic stress; PTG, post-traumatic growth

*Note*: Both models adjusted for housing status in 2013 and baseline covariates (2010): sex, age, educational attainment, working status, equivalized household income, divorced/bereavement, depressive symptoms (≥5 points of Geriatric Depression Scale-15), smoking and drinking alcohol.

Table 3 shows the results of linear regression models (models 1 and 2) and marginal structural models considering time-dependent confounding (model 3). Severely affected PTS was associated with higher PTG scores in all models, including those considering baseline covariates (coefficient 0.38, 95% CI 0.28, 0.48, *P* < 0.01 in model 1), time-series covariates (coefficient 0.33, 95% CI 0.23, 0.43, *P* < 0.01 in model 2) and time-dependent confounders using inverse probability weighting (coefficient 0.35, 95% CI 0.24, 0.46, *P* < 0.01 in model 3).

**Table 3.** Association between PTS and PTG using five-wave panel data

	Model 1: adjusted for baseline covariates		Model 2: adjusted for time-series covariates		Model 3: using IPW (marginal structural model)	
	Coefficient (95% CI)	*P*	Coefficient (95% CI)	*P*	Coefficient (95% CI)	*P*
Severely affected PTS (≥6 points of SQD score)	0.38 (0.28, 0.48)	<0.01	0.33 (0.23, 0.43)	<0.01	0.35 (0.24, 0.46)	<0.01
Baseline characteristics						
Age	−0.01 (−0.01, 0.01)	0.33	−0.01 (−0.01, 0.01)	0.09	0.01 (−0.01, 0.01)	<0.01
Sex	0.01 (−0.06, 0.07)	0.83	−0.07 (−0.13, −0.02)	0.01	−0.08 (−0.12, −0.04)	<0.01
Education	0.01 (−0.03, 0.04)	0.66	0.01 (−0.02, 0.05)	0.40	0.02 (−0.01, 0.03)	0.11
Equivalized household income	−0.01 (−0.01, 0.01)	0.42	−0.01 (−0.01, 0.01)	0.70	−0.01 (−0.01, 0.01)	0.67
Divorce/bereavement	0.01 (−0.06, 0.07)	0.85	0.01 (−0.06, 0.07)	0.92	0.01 (−0.03, 0.04)	0.72
Working	0.03 (−0.03, 0.09)	0.39	0.03 (−0.02, 0.09)	0.25	0.03 (−0.01, 0.07)	0.06
The number of adverse life events	0.01 (−0.02, 0.04)	0.41	0.01 (−0.02, 0.04)	0.48	0.01 (−0.01, 0.03)	0.33
Severe depressive symptoms (≥5 points of GDS-15)	−0.07 (−0.13, −0.01)	0.03				
Current smoking	−0.02 (−0.10, 0.06)	0.66				
Current drinking	−0.01 (−0.06, 0.06)	0.98				
Time-dependent covariates						
Severe depressive symptoms (≥5 points of GDS-15)			0.04 (−0.03, 0.10)	0.30	0.06 (−0.01, 0.12)	0.07
Current smoking			−0.17 (−0.26, −0.07)	<0.01	−0.17 (−0.23, −0.11)	<0.01
Current drinking			−0.13 (−0.19, −0.07)	<0.01	−0.14 (−0.18, −0.09)	<0.01
Housing status after the disaster (ref. no relocation)						
Government-provided housing			0.02 (−0.17, 0.21)	0.84	0.01 (−0.13, 0.13)	0.97
Apartment in the open-rental market			0.07 (−0.20, 0.33)	0.62	0.06 (−0.14, 0.25)	0.56
Newly purchased housing			0.34 (0.19, 0.50)	<0.01	0.38 (0.26, 0.49)	<0.01
Cons.	1.59 (1.15, 2.03)	<0.01	1.86 (1.43, 2.29)	<0.01	1.86 (1.60, 2.11)	<0.01

Abbreviations: Cons, constant; CI, confidence interval; PTS, post-traumatic stress; PTG, post-traumatic growth; IPW, inverse probability weighting

We also utilized imbalanced data consisting of baseline respondents who participated in at least one of the follow-up surveys (Table 4). We considered attrition during the follow-up period as well as time-dependent confounding, using inverse probability weighting. The results also showed that severely affected PTS was related to higher scores of PTG in all models which considered baseline covariates (coefficient 0.17, 95% CI 0.12, 0.22, *P* < 0.01 in model 1), time-series covariates (coefficient 0.12, 95% CI 0.06, 0.17, *P* < 0.01 in model 2) and time-dependent confounders using inverse probability weighting (coefficient 0.16, 95% CI 0.09, 0.24, *P* < 0.01 in model 3).

**Table 4.** Association between PTS and PTG using the imbalanced data

	Model 1: adjusted for baseline covariates		Model 2: adjusted for time-series covariates		Model 3: using IPW (marginal structural model)	
	Coefficient (95% CI)	*P*	Coefficient (95% CI)	*P*	Coefficient (95% CI)	*P*
Severely affected PTS (≥6 points of SQD score)	0.17 (0.12, 0.22)	<0.01	0.12 (0.06, 0.17)	<0.01	0.16 (0.09, 0.24)	<0.01
Baseline characteristics						
Age	−0.01 (−0.01, −0.01)	<0.01	−0.01 (−0.01, −0.01)	<0.01	−0.01 (−0.01, −0.01)	<0.01
Sex	−0.01 (−0.04, 0.04)	0.97	−0.01 (−0.04, 0.02)	0.68	0.02 (−0.01, 0.05)	0.19
Education	0.02 (0.01, 0.04)	0.02	0.02 (0.01, 0.04)	0.03	0.02 (0.01, 0.03)	0.02
Equivalized household income	0.01 (−0.01, 0.01)	0.17	0.01 (−0.01, 0.01)	0.10	0.01 (−0.01, 0.01)	0.06
Divorce/bereavement	−0.01 (−0.04, 0.03)	0.74	−0.01 (−0.04, 0.03)	0.75	−0.01 (−0.03, 0.02)	0.88
Working	0.02 (−0.01, 0.06)	0.18	0.03 (−0.01, 0.06)	0.09	0.03 (0.01, 0.06)	0.03
The number of adverse life events	0.01 (−0.01, 0.02)	0.37	0.01 (−0.01, 0.02)	0.59	−0.01 (−0.01, 0.02)	0.68
Severe depressive symptoms (≥5 points of GDS−15)	−0.07 (−0.11, −0.04)	<0.01				
Current smoking	0.02 (−0.01, 0.06)	0.17				
Current drinking	0.01 (−0.02, 0.04)	0.52				
Time-dependent covariates						
Severe depressive symptoms (≥5 points of GDS-15)			−0.02 (−0.05, 0.02)	0.35	−0.01 (−0.04, 0.02)	0.49
Current smoking			−0.23 (−0.26, −0.19)	<0.01	−0.23 (−0.25, −0.21)	<0.01
Current drinking			−0.08 (−0.11, −0.05)	<0.01	−0.07 (−0.09, −0.04)	<0.01
Housing status after the disaster (ref. no relocation)						
Government-provided housing			−0.19 (−0.24, −0.13)	<0.01	−0.18 (−0.22, −0.13)	<0.01
Apartment in the open-rental market			−0.02 (−0.16, 0.12)	0.80	−0.03 (−0.18, 0.13)	0.72
Newly purchased housing			0.35 (0.25, 0.45)	<0.01	0.29 (0.17, 0.41)	<0.01
Cons.	2.02 (1.81, 2.22)	<0.01	2.10 (1.90, 2.30)	<0.01	1.94 (1.78, 2.10)	<0.01

Abbreviations: Cons, constant; CI, confidence interval; PTS, post-traumatic stress; PTG, post-traumatic growth; IPW, inverse probability weighting

**Figure 2.**
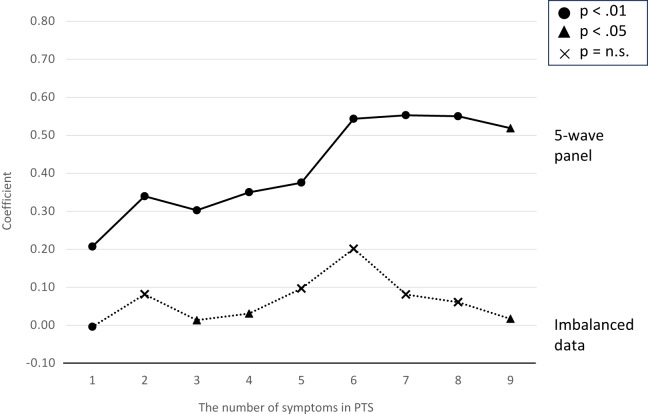
Association between ordinal variables of the PTS score and PTG: results of marginal structural models in both data.

In the further analysis, we utilized the total PTS scores as an ordinal scale. The results presented that PTS scores had an inverse U-shaped association with PTG in both datasets (Fig. 2).

In the sensitivity analysis, we also utilized a continuous variable for the PTS score. In both data sets, the continuous PTS score was associated with PTG when adjusting for baseline covariates and time-series confounders, while the results from the marginal structural models did not exhibit a significant association with PTG (Tables S2 and S3).

## Discussion

This study had two aims: (1) to explore the associations of PTS and PTG with trauma-related diseases that were objectively assessed and (2) to examine the association between PTS and PTG using marginal structural models to address time-dependent confounding.

First, some research has suggested that PTG might be a form of cognitive bias among survivors – for instance, social desirability bias, positive attrition bias or the illusory perception of personal growth as a compensatory response to trauma (Gower *et al.*, 2022). As shown in Tables 3 and 4, a high risk of depressive symptoms was not significantly associated with PTG, while severely affected PTS was linked to higher scores of PTG. This result implies that post-disaster psychopathology exhibits inconsistent associations with PTG, indicating that PTG should not be seen as a justification for the struggles associated with depressive symptoms. Furthermore, we added evidence suggesting that PTG may not merely be an illusory perception of personal growth. As depicted in the updated Table 2, PTG was found to be protectively associated with functional disability and cognitive decline, as assessed by trained investigators and physicians, whereas PTS showed no significant associations with these outcomes. This differential association between PTS and PTG with objective assessment outcomes implies that PTG is more than just a cognitive bias. Further research is needed to explore the unique relationship between PTG and health outcomes.

Second, severely affected PTS was associated with higher scores of PTG, even after adjusting for depressive symptoms and substance use (i.e., smoking and drinking) as comorbidities of PTS. The time-series variables of drinking and smoking habits were associated with lower levels of PTG. However, the time-series variable of depressive symptoms was not associated with PTG, while pre-disaster depressive symptoms were linked to higher scores of PTG. These variables may have mutually influenced each other over time and subsequently led to the achievement of PTG. For instance, respondents with pre-disaster depressive symptoms (t_0_) may have implemented ineffective stress coping strategies, such as substance use as avoidance coping, in the aftermath of the disaster (Orzechowska *et al.*, 2022, 2013), resulting in an increased risk of severe PTS with comorbidities (t_1_). Nicotine dependence could aggravate PTS conditions in the same survey wave (t_1_) by producing dysregulation of the hypothalamus–pituitary–adrenal system (Koenen *et al.*, 2005). Excessive drinking would also reinforce symptoms of PTS (t_1_), because it enhances the activity of an inhibition neurotransmitter (e.g., gamma-aminobutyric acid) in the brain region linked to anxiety production (Stewart, 1996). PTS could worsen the substance use condition in the subsequent wave (t_2_), which may affect the level of PTG in a further wave (t_4_) due to avoidance coping being linked to the lack of cognitive processing needed to reconstruct their identity and world assumptions (Stump and Smith, 2008).

Furthermore, after controlling for time-dependent confounding, an ordinal variable of the PTS score appeared to exhibit an inverse U-shaped association with PTG. This result aligns with the non-significant linear relationship between PTS and PTG when using the PTS score as a continuous variable. Our findings provided robust support for the theory of PTG, suggesting that moderate levels of psychological struggles (i.e., PTS) are essential for achieving PTG, whereas intense PTS may hinder the attainment of PTG. A plausible mechanism may explain the inverse U-shaped association. Victims with PTS suffer intrusive thoughts about traumatic experiences that evoke negative emotions. Respondents exhibiting severe PTS might not have found benefits and meaning in their disaster experiences due to persistent uncontrollable thoughts. Moreover, their comorbidities also have aggravated the recovery progress from severe PTS. On the other hand, deliberate rumination may enhance the understanding of the meaning of traumatic events and the reconstruction of world assumptions, leading to PTG (Wang *et al.*, 2020). Recent research has indicated that social support is positively associated with deliberate rumination and inversely associated with intrusive thoughts among patients with oesophageal cancer (Li *et al.*, 2022). Therefore, interventions encouraging social support might be beneficial in attaining PTG by facilitating deliberate rumination.

We also demonstrated that purchasing new housing after the disaster was associated with higher scores of PTG in both the five-wave panel and imbalanced datasets. We previously showed that participants who moved into new private housing by the 2016 survey were less likely to have severely affected depressive symptoms (Hikichi *et al.*, 2021). Their higher socioeconomic status, enabling them to afford new housing, might have contributed to heightened PTG.

A major strength of the present study is the availability of pre-disaster information. Baseline depressive symptoms are also associated with severely affected PTS (see Table S4) and lower scores of PTG (see Tables 3 and 4). Therefore, pre-disaster depressive symptoms could be an important confounder in the association between PTS and PTG, which may have led to the null association observed in previous studies.

This study has some limitations. First, survivor bias might have occurred because participants who suffered from severe PTS might have dropped out by the 2022 survey. However, we found similar results even when we used the imbalanced data including dropped cases. Second, the one-time assessment of PTG could lead to an underestimation of the associations between PTS, its comorbidities, and PTG. According to a systematic review finding, victims of natural disasters can achieve PTG within a few years after the event (Amiri *et al.*, 2021). However, we did not measure PTG until the 2022 survey. Therefore, the association between PTS and PTG might have been underestimated compared to a hypothetical scenario where PTG had been measured since the 2013 survey. Third, we were unable to use pre-disaster information on PTS, although we adjusted for baseline adverse life events in the analyses. Fourth, a lack of clinical assessment of PTS and depressive symptoms could lead to misclassification of these mental diseases, even though we used clinically validated scales. Fifth, we did not measure the quantity of smoking and alcohol consumption, which could lead to an oversight of individual differences in these behaviours. Sixth, we might not have ruled out the possibility of an illusionary perception of personal growth as a compensatory response to traumatic experiences. Finally, the lack of post-disaster adverse life events could be a residual confounding factor, resulting in biased estimations.

In conclusion, we demonstrated that PTG and PTS were differentially associated with functional and cognitive disabilities. Thus, PTG might not simply be a cognitive bias among survivors with severe PTS. Severely affected PTS was associated with higher scores of PTG, even after adjusting for time-dependent confounders. The results also indicated that the number of PTS symptoms had an inverse U-shaped association with PTG. Interventions that encourage social support could be beneficial in achieving PTG by facilitating deliberate rumination.

## Supporting information

Hikichi et al. supplementary material Hikichi et al. supplementary material

## Data Availability

All data needed to evaluate the conclusions in the paper are present in the paper and/or the Supplementary Materials. The JAGES data used in this study will be made available upon request, as per NIH data access policies. The authors require the applicant to submit an analysis proposal to be reviewed by an internal JAGES committee to avoid duplication. Confidentiality concerns prevent us from depositing our data in a public repository. Authors requesting access to the Iwanuma data need to contact the principal investigator of the parent cohort (K.K.) and the Iwanuma sub-study principal investigator (I.K.) in writing. Proposals submitted by outside investigators will be discussed during the monthly investigators’ meeting to ensure that there is no overlap with ongoing analyses. If approval to access the data is granted, the JAGES researchers will request the outside investigator to help financially support our data manager’s time to prepare the data for outside use.

## Supplementary material

The supplementary material for this article can be found at https://doi.org/10.1017/S2045796024000362.

## References

[ref1] Aldrich N and Benson WF (2008) Disaster preparedness and the chronic disease needs of vulnerable older adults. *Preventing Chronic Disease* 5(1), A27. PMC2248769 18082016

[ref2] Amiri H, Nakhaee N, Nagyova I, Timkova V, Okhovati M, Nekoei-Moghadam M and Zahedi R (2021) Posttraumatic growth after earthquake: A systematic review and meta-analysis. *The International Journal of Social Psychiatry* 67(7), 867–877. 33611959 10.1177/0020764021995856

[ref3] Blake DD, Weathers FW, Nagy LM, Kaloupek DG, Gusman FD, Charney DS and Keane TM (1995) The development of a Clinician-Administered PTSD Scale. *Journal of Traumatic Stress* 8(1), 75–90. 7712061 10.1007/BF02105408

[ref4] Flory JD and Yehuda R (2015) Comorbidity between post-traumatic stress disorder and major depressive disorder: Alternative explanations and treatment considerations. *Dialogues in Clinical Neuroscience* 17(2), 141–150. 26246789 10.31887/DCNS.2015.17.2/jflory PMC4518698

[ref5] Fraus K, Dominick W, Walenski A and Taku K (2023) The impact of multiple stressful life events on posttraumatic growth in adolescence. *Psychological Trauma: Theory, Research, Practice, and Policy* 15(1), 10–17. 34766804 10.1037/tra0001181

[ref6] Fujii S, Kato H and Maeda K (2007) A simple interview-format screening measure for disaster mental health: An instrument newly developed after the 1995 Great Hanshin Earthquake in Japan-the Screening Questionnaire for Disaster Mental Health (SQD). *Kobe Journal of Medical Sciences* 53(6), 375–385. 18762732

[ref7] Godin O, Elbejjani M and Kaufman JS (2012) Body mass index, blood pressure, and risk of depression in the elderly: A marginal structural model. *American Journal of Epidemiology* 176(3), 204–213. 22781426 10.1093/aje/kws003

[ref8] Gower T, Pham J, Jouriles EN, Rosenfield D and Bowen HJ (2022) Cognitive biases in perceptions of posttraumatic growth: A systematic review and meta-analysis. *Clinical Psychology Review* 94, 102159. 10.1016/j.cpr.2022.102159 35483274

[ref9] Greenblatt-Kimron L (2021) World assumptions and post-traumatic growth among older adults: The case of Holocaust survivors. *Stress and Health* 37(2), 353–363. 33098210 10.1002/smi.3000

[ref10] Hikichi H, Aida J, Kondo K and Kawachi I (2021) Six-year follow-up study of residential displacement and health outcomes following the 2011 Japan Earthquake and Tsunami. *Proceedings of the National Academy of Sciences* 118(2), e2014226118. 10.1073/pnas.2014226118 PMC7812822 33397722

[ref11] Hikichi H, Aida J, Kondo K, Tsuboya T, Matsuyama Y, Subramanian SV and Kawachi I (2016) Increased risk of dementia in the aftermath of the 2011 Great East Japan Earthquake and Tsunami. *Proceedings of the National Academy of Sciences* 113(45), E6911–E6918. 10.1073/pnas.1607793113 PMC5111665 27791093

[ref12] Kadri A, Gracey F and Leddy A (2022) What factors are associated with posttraumatic growth in older adults? A systematic review. *Clinical Gerontologist* 9, 1–18. 10.1080/07317115.2022.2034200 35138231

[ref13] Koenen KC, Hitsman B, Lyons MJ, Niaura R, McCaffery J, Goldberg J, Eisen SA, True W and Tsuang M (2005) A twin registry study of the relationship between posttraumatic stress disorder and nicotine dependence in men. *Archives of General Psychiatry* 62(11), 1258–1265. 16275813 10.1001/archpsyc.62.11.1258

[ref14] Li J, Xue L and Pan H (2022) Social support and spiritual well-being of patients with esophageal cancer aged over 50 Years: The mediating role of rumination. *Frontiers in Psychiatry* 13, 805380. 10.3389/fpsyt.2022.805380 PMC8931259 35308890

[ref15] Long LJ, Phillips CA, Glover N, Richardson AL, D’Souza JM, Cunningham-Erdogdu P and Gallagher MW (2021) A meta-analytic review of the relationship between posttraumatic growth, anxiety, and depression. *Journal of Happiness Studies* 22(8), 3703–3728.

[ref16] Mansournia MA, Etminan M, Danaei G, Kaufman JS and Collins G (2017) Handling time varying confounding in observational research. *British Medical Journal* 359, j4587. 10.1136/bmj.j4587 29038130

[ref17] Olivares-Tirado P and Tamiya N (2014) Development of the Long-Term Care Insurance System in Japan. In Olivares-Tirado P and Tamiya N (eds), *Trends and Factors in Japan’s Long-Term Care Insurance System*. Netherlands: Springer, 15–42.

[ref18] Organisation for Economic Co-operation and Development (OECD) (2013) *OECD Framework for Statistics on the Distribution of Household Income, Consumption and Wealth*. Paris: OECD Publishing.

[ref19] Orzechowska A, Bliźniewska-Kowalska K, Gałecki P, Szulc A, Płaza O, Su KP, Georgescu D and Gałecka M (2022) Ways of coping with stress among patients with depressive disorders. *Journal of Clinical Medicine* 11(21), 6500. 10.3390/jcm11216500 PMC9653687 36362729

[ref20] Orzechowska A, Zajączkowska M, Talarowska M and Gałecki P (2013) Depression and ways of coping with stress: A preliminary study. *Medical Science Monitor: International Medical Journal of Experimental and Clinical Research* 19, 1050–1056. 24270182 10.12659/MSM.889778 PMC3852369

[ref21] Oshiro R, Soejima T, Kita S, Benson K, Kibi S, Hiraki K, Kamibeppu K and Taku K (2023) Reliability and Validity of the Japanese Version of the Short Form of the Expanded Version of the Posttraumatic Growth Inventory (PTGI-X-SF-J): A Cross-Sectional Study. *International Journal of Environmental Research & Public Health* 20(11), 5965. 10.3390/ijerph20115965 PMC10252444 37297569

[ref22] Robins JM, Hern MA and Brumback B (2000) Marginal structural models and causal inference in epidemiology. *Epidemiology* 11(5), 550–560. 10955408 10.1097/00001648-200009000-00011

[ref23] Santos-Eggimann B, Cuénoud P, Spagnoli J and Junod J (2009) Prevalence of frailty in middle-aged and older community-dwelling Europeans living in 10 countries. *The Journals of Gerontology Series A: Biological Sciences and Medical Sciences* 64(6), 675–681. 19276189 10.1093/gerona/glp012 PMC2800805

[ref24] Sareen J (2014) Posttraumatic stress disorder in adults: Impact, comorbidity, risk factors, and treatment. *Canadian Journal of Psychiatry* 59(9), 460–467. 25565692 10.1177/070674371405900902 PMC4168808

[ref25] Shakespeare-Finch J and Lurie-Beck J (2014) A meta-analytic clarification of the relationship between posttraumatic growth and symptoms of posttraumatic distress disorder. *Journal of Anxiety Disorders* 28(2), 223–229. 24291397 10.1016/j.janxdis.2013.10.005

[ref26] Shiba K, Hikichi H, Okuzono SS, VanderWeele TJ, Arcaya M, Daoud A, Cowden RG, Yazawa A, Zhu DT, Aida J, Kondo K and Kawachi I (2022) Long-term associations between disaster-related home loss and health and well-being of older survivors: Nine years after the 2011 Great East Japan Earthquake and Tsunami. *Environmental Health Perspectives* 130(7), 77001. 10.1289/EHP10903 PMC9249145 35776697

[ref27] Stewart SH (1996) Alcohol abuse in individuals exposed to trauma: A critical review. *Psychological Bulletin* 120(1), 83–112. 8711018 10.1037/0033-2909.120.1.83

[ref28] Stump MJ and Smith JE (2008) The relationship between posttraumatic growth and substance use in homeless women with histories of traumatic experience. *The American Journal on Addictions* 17(6), 478–487. 19034739 10.1080/10550490802409017

[ref29] Sugishita K, Sugishita M, Hemmi I, Asada T and Tanigawa T (2017) A validity and reliability study of the Japanese version of the Geriatric Depression Scale 15 (GDS-15-J). *Clinical Gerontologist* 40(4), 233–240. 28452641 10.1080/07317115.2016.1199452

[ref30] Tamiya N, Noguchi H, Nishi A, Reich MR, Ikegami N, Hashimoto H, Shibuya K, Kawachi I and Campbell JC (2011) Population ageing and wellbeing: Lessons from Japan’s long-term care insurance policy. *The Lancet* 378(9797), 1183–1192. 10.1016/S0140-6736(11)61176-8 21885099

[ref31] Tedeschi RG and Calhoun LG (2004) Posttraumatic growth: Conceptual foundations and empirical evidence. *Psychological Inquiry* 15(1), 1–18.

[ref32] Wang W, Wu X and Lan X (2020) Rumination mediates the relationships of fear and guilt to posttraumatic stress disorder and posttraumatic growth among adolescents after the Ya’an earthquake. *European Journal of Psychotraumatology* 11(1), 1704993. 10.1080/20008198.2019.1704993 PMC6968513 32002139

[ref33] Weintraub D, Oehlberg KA, Katz IR and Stern MB (2006) Test characteristics of the 15-item geriatric depression scale and Hamilton depression rating scale in Parkinson disease. *American Journal of Geriatric Psychiatry* 14(2), 169–175. 10.1097/01.JGP.0000192488.66049.4b PMC1571046 16473982

