# Supplementary material for: Longitudinal associations between post-traumatic stress and post-traumatic growth among older adults 11 years after a disaster
Source: Epidemiol Psychiatr Sci. 2024 Jun 26;33:e33. doi: 10.1017/S2045796024000362 (PMC11362680; doi:10.1017/S2045796024000362)
Supplement: Hikichi et al. supplementary material [file S2045796024000362sup001.pdf]

Supplementary Information

Longitudinal associations between posttraumatic stress and posttraumatic growth among older adults 11 years after a disaster

Authors: Hiroyuki Hikichi, PhD; Kanako Taku, PhD; Jun Aida, DDS, PhD; Katsunori Kondo, MD, PhD; and Ichiro Kawachi, MD, PhD

Corresponding author: Hiroyuki Hikichi, PhD

Email: hikichi@med.kitasato-u.ac.jp

Figure S1. Causal diagram representing time-dependent confounding

Figure S2. Map of the tsunami-inundated area in Iwanuma City, Japan

Table S1. Proportions of comorbidities of severely affected versus slightly/moderately affected PTS in each follow-up survey

Figure S3. The result of confirmatory factor analysis for posttraumatic growth

Table S2. Associations between PTS (continuous variable) and PTG using 5-wave panel data

Table S3. Associations between PTS (continuous variable) and PTG using imbalanced data

Table S4. An association between baseline depressive symptoms and PTS

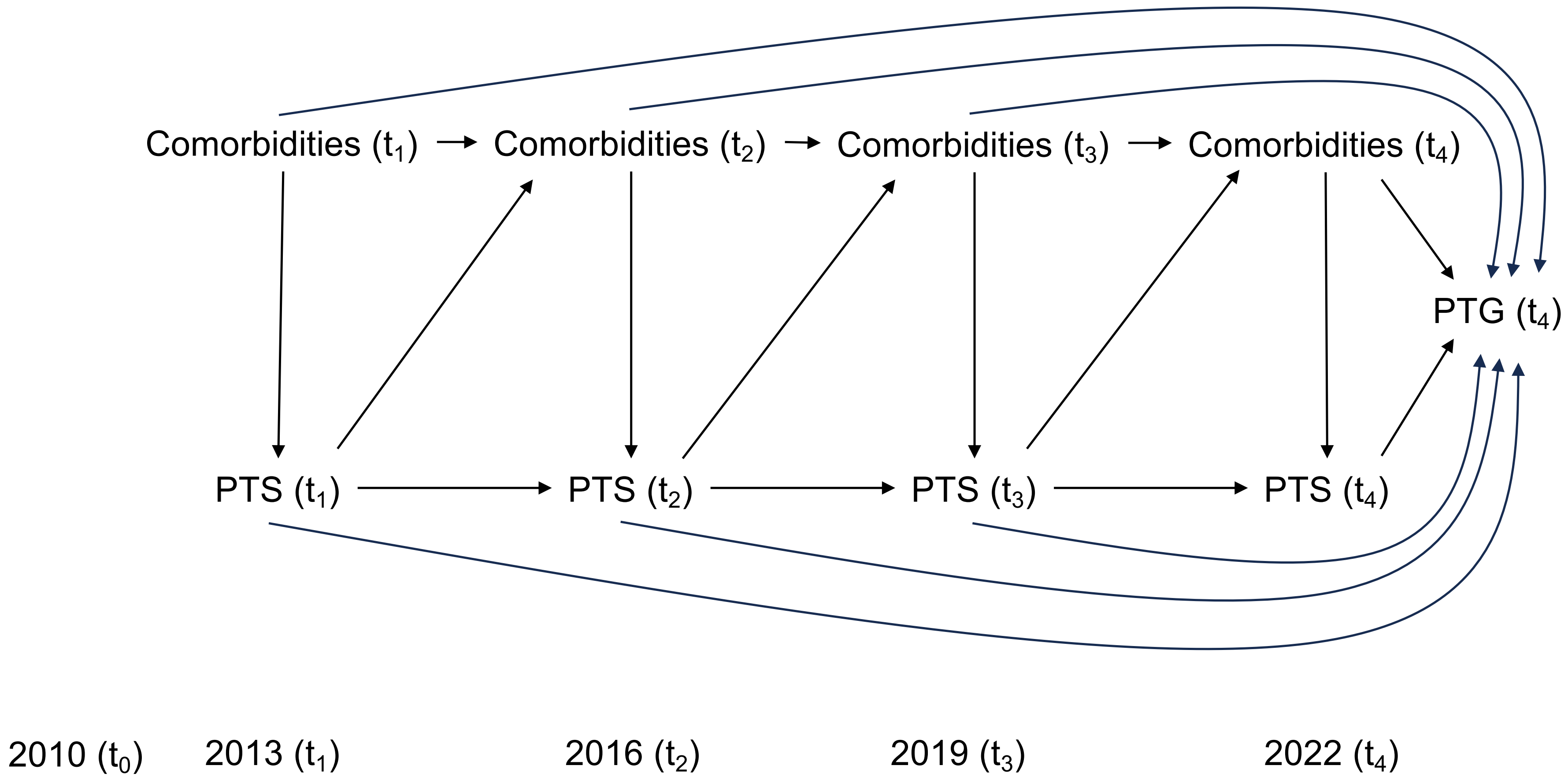

Figure S1. Causal diagram representing time-dependent confounding

Note: Comorbidities of PTS consist of depressive symptoms, and smoking and drinking habits.
Abbreviation: PTS, Posttraumatic stress; PTG, Posttraumatic growth

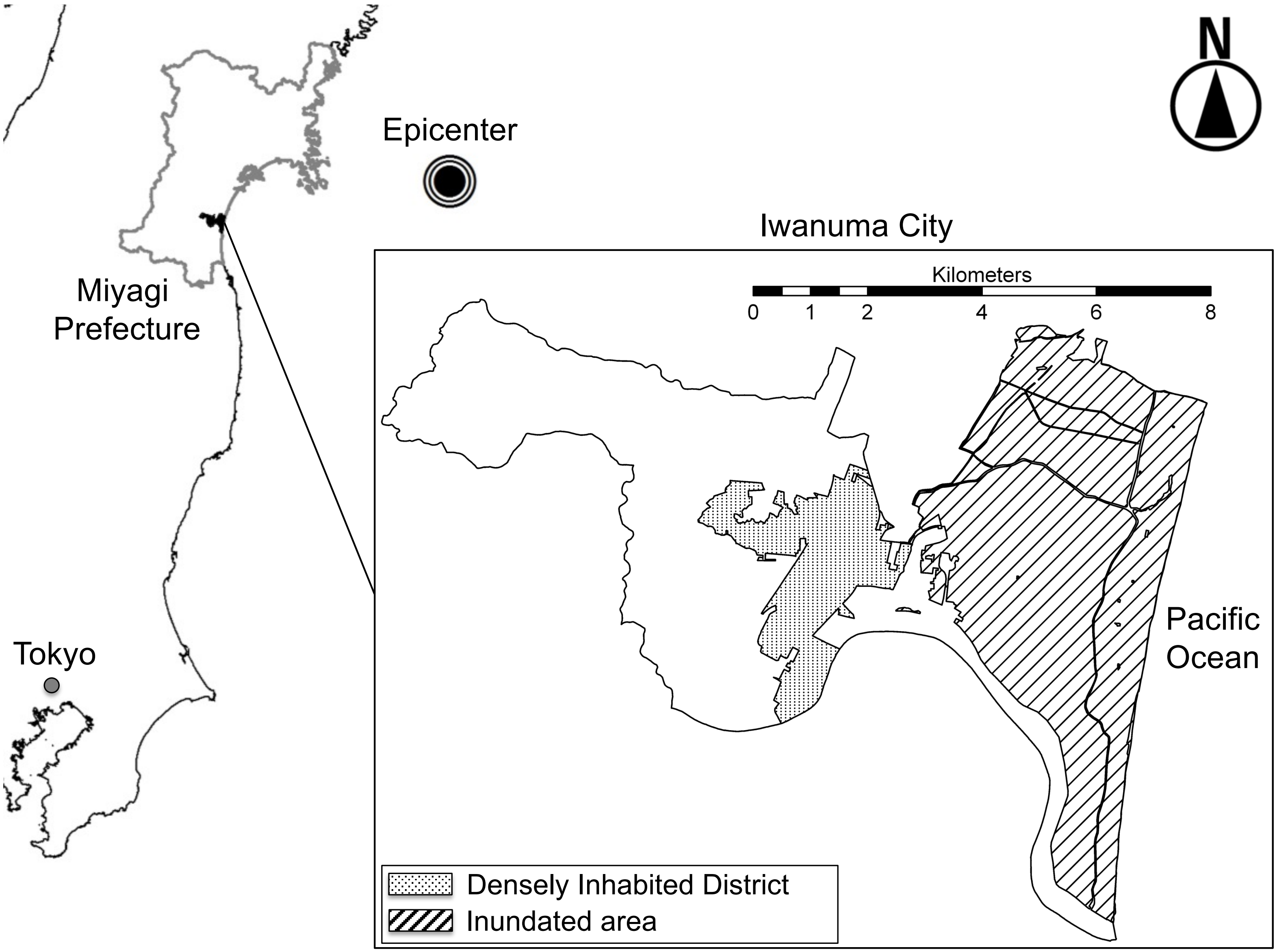

Figure S2. Map of the tsunami-inundated area in Iwanuma City, Japan

Table S1. Proportions of comorbidities of severely affected versus slightly/moderately affected PTS in each follow-up survey

	Severe depressive symptoms (%)		Smoker (%)		Drinker (%)	
Year	Slight/moderate PTS	Severe PTS	Slight/moderate PTS	Severe PTS	Slight/moderate PTS	Severe PTS
2013	20.5	48.4	8.6	5.6	38.3	21.4
2016	16.9	35.6	5.9	4.8	37.8	26.9
2019	21.1	50.4	5.0	5.4	35.1	22.5
2022	24.7	51.1	4.4	5.0	32.1	25.9

Note: Severely affected PTS was defined as a score of ≥ 6 points on the Screening Questionnaire for Disaster-Related Mental Health. Severe depressive symptoms were defined as a score of ≥ 5 points on the Geriatric Depression Scale-15.

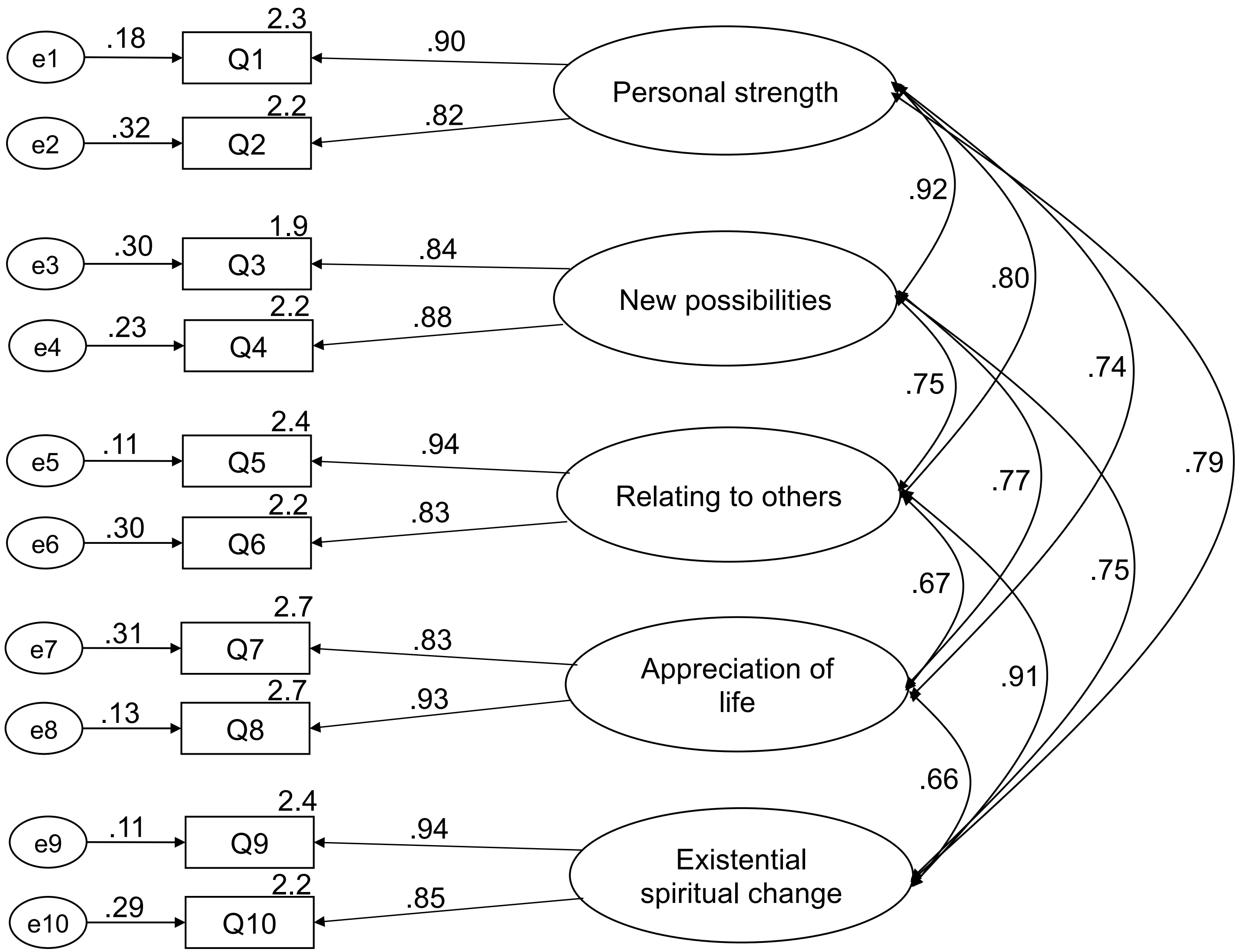

Figure S3. The result of confirmatory factor analysis for posttraumatic growth

Note: The goodness of fit indices: comparative fit index (CFI) 0.981, root-mean-square error of approximation (RMSEA) 0.079, and standardized-root-mean-square residual (SRMA) 0.025

Table S2. Associations between PTS (continuous variable) and PTG using 5-wave panel data

	Model 1: adjusted for baseline covariates		Model 2: adjusted for time-series covariates		Model 3: using IPW (marginal structural model)	
	Coefficient (95% CI)	p	Coefficient (95% CI)	p	Coefficient (95% CI)	p
PTS	0.02 (0.01, 0.03)	< .01	0.01 (0.01, 0.02)	< .01	0.01 (-0.01, 0.02)	.07
Baseline characteristics						
Age	-0.02 (-0.02, -0.01)	< .01	-0.02 (-0.02, -0.01)	< .01	-0.02 (-0.02, -0.01)	< .01
Sex	-0.01 (-0.06, 0.05)	.79	-0.04 (-0.09, -0.01)	.04	-0.03 (-0.06, 0.01)	.14
Education	0.03 (0.01, 0.05)	.03	0.03 (0.01, 0.05)	.04	0.04 (0.02, 0.06)	< .01
Equivalized household income (10,000JPY)	0.01 (-0.01, 0.01)	.24	0.01 (-0.01, 0.01)	.10	0.01 (-0.01, 0.01)	.05
Divorce/bereavement	-0.01 (-0.05, 0.04)	.88	-0.01 (-0.05, 0.04)	.73	-0.01 (-0.05, 0.02)	.54
Working	0.03 (-0.02, 0.07)	.23	0.04 (-0.01, 0.08)	.12	0.03 (-0.01, 0.07)	.07
The number of adverse life events	0.01 (-0.01, 0.03)	.37	0.01 (-0.02, 0.03)	.75	0.01 (-0.02, 0.03)	.57
Depressive symptoms [a]	-0.01 (-0.02, -0.01)	< .01				
Current smoking	0.03 (-0.02, 0.08)	.22				
Current drinking	0.01 (-0.03, 0.05)	.68				
Time-dependent covariates						
Severe depressive symptoms [a]			0.01 (-0.01, 0.01)	.42	0.01 (-0.01, 0.01)	.15
Current smoking			-0.11 (-0.18, -0.04)	< .01	-0.12 (-0.17, -0.06)	< .01
Current drinking			-0.09 (-0.13, -0.05)	< .01	-0.07 (-0.11, -0.04)	< .01
Post-disaster housing (ref.: no relocation)						
Government-provided housing			-0.24 (-0.30, -0.17)	< .01	-0.23 (-0.27, -0.19)	< .01
Apartment in the open-rental market			-0.08 (-0.24, 0.08)	.32	-0.07 (-0.21, 0.07)	.33
Newly purchased housing			0.30 (0.19, 0.42)	< .01	0.35 (0.24, 0.46)	< .01
Cons.	2.32 (2.04, 2.60)	< .01	2.44 (2.16, 2.71)	< .01	2.36 (2.14, 2.58)	< .01

Abbreviation: Cons, constant; CI, Confidence interval; PTS, Posttraumatic stress; PTG, Posttraumatic growth; IPW, inverse probability weighting

[a] ≧ 5 p of GDS-15

Table S3. Associations between PTS (continuous variable) and PTG using imbalanced data

	Model 1: adjusted for baseline covariates		Model 2: adjusted for time-series covariates		Model 3: using IPW (marginal structural model)	
	Coefficient (95% CI)	p	Coefficient (95% CI)	p	Coefficient (95% CI)	p
PTS	0.02 (0.01, 0.03)	< .01	0.01 (0.01, 0.02)	< .01	0.01 (-0.01, 0.02)	.05
Baseline characteristics						
Age	-0.02 (-0.02, -0.01)	< .01	-0.02 (-0.02, -0.01)	< .01	-0.01 (-0.02, -0.01)	< .01
Sex	-0.01 (-0.06, 0.05)	.79	-0.04 (-0.09, 0.01)	.04	-0.01 (-0.04, 0.03)	.78
Education	0.03 (0.01, 0.05)	.03	0.03 (0.01, 0.05)	.04	0.04 (0.02, 0.06)	< .01
Equivalized household income (10,000JPY)	0.01 (-0.01, 0.01)	.24	0.01 (-0.01, 0.01)	.10	0.01 (-0.01, 0.01)	.22
Divorce/bereavement	-0.01 (-0.05, 0.04)	.88	-0.01 (-0.05, 0.04)	.73	-0.01 (-0.04, 0.03)	.83
Working	0.03 (-0.02, 0.07)	.23	0.04 (-0.01, 0.08)	.12	0.03 (-0.01, 0.08)	.11
The number of adverse life events	0.01 (-0.01, 0.03)	.37	0.01 (-0.02, 0.03)	.75	0.01 (-0.02, 0.03)	.83
Depressive symptoms [a]	-0.01 (-0.02, -0.01)	< .01				
Current smoking	0.03 (-0.02, 0.08)	.22				
Current drinking	0.01 (-0.03, 0.05)	.68				
Time-dependent covariates						
Severe depressive symptoms [a]			0.01 (-0.01, 0.01)	.42	0.01 (-0.01, 0.01)	.21
Current smoking			-0.11 (-0.18, -0.04)	< .01	-0.15 (-0.21, -0.10)	< .01
Current drinking			-0.09 (-0.13, -0.05)	< .01	-0.05 (-0.09, -0.01)	.02
Post-disaster housing (ref.: no relocation)						
Government-provided housing			-0.24 (-0.30, -0.17)	< .01	-0.21 (-0.25, -0.17)	< .01
Apartment in the open-rental market			-0.08 (-0.24, 0.08)	.32	-0.09 (-0.23, 0.05)	.21
Newly purchased housing			0.30 (0.19, 0.42)	< .01	0.27 (0.15, 0.40)	< .01
Cons.	2.32 (2.04, 2.60)	< .01	2.44 (2.16, 2.71)	< .01	2.21 (1.98, 2.45)	< .01

Abbreviation: Cons, constant; CI, Confidence interval; PTS, Posttraumatic stress; PTG, Posttraumatic growth; IPW, inverse probability weighting

[a] ≧ 5 p of GDS-15

Table S4. An association between baseline depressive symptoms and PTS (binary variable)

	Odds (95% CI)	p
Baseline severe depressive symptoms (≧ 5 p of GDS-15) [a]	2.42 (1.94, 3.02)	< .01
Baseline characteristics		
Age	1.02 (0.99, 1.04)	.06
Sex	1.52 (1.18, 1.96)	< .01
Education	0.80 (0.70, 0.92)	< .01
Equivalized household income	0.99 (0.98, 0.99)	< .01
Divorce/bereavement	0.99 (0.78, 1.26)	.94
Working	1.07 (0.83, 1.38)	.60
The number of adverse life events	1.12 (0.99, 1.25)	.06
Current smoking	0.82 (0.57, 1.19)	.30
Current drinking	0.83 (0.65, 1.06)	.13
Cons.	0.02 (0.01, 0.11)	< .01

Abbreviation: Cons, constant; CI, Confidence interval; PTS, Posttraumatic stress symptoms; GDS, Geriatric Depression Scale
[a] ≧ 5 p of GDS-15
